# Exploring virus presence in field-collected potato leaf samples using RNA sequencing

**DOI:** 10.1186/s43141-023-00561-2

**Published:** 2023-10-20

**Authors:** Esraa A. Elwan, Mona Rabie, Engy E. Abdel Aleem, Faiza A. Fattouh, Meenakshi S. Kagda, Heba A. H. Zaghloul

**Affiliations:** 1 Department of Botany and Microbiology, Faculty of Science, Alexandria University, Alexandria, 21511 Egypt; 2 Genetics Department, School of Medicine, Stanford University, Stanford, CA USA

**Keywords:** Virome, RNA-Seq, Potato viruses, Alfalfa mosaic virus, Potato leafroll virus

## Abstract

**Background:**

The quick and accurate identification of viruses is essential for plant disease management. Next-generation sequencing (NGS) technology may allow the discovery, detection, and identification of plant pathogens. This study adopted RNA-sequencing (RNA-Seq) technology to explore the viruses in three potato plants (S3, S4, and S6) growing under field conditions.

**Results:**

Potato-known infecting viruses, such as alfalfa mosaic virus (AMV), potato leafroll virus (PLRV), and potato virus Y (PVY), were identified using bioinformatics programs and validated using RT-PCR. The presence of these potato viruses was also confirmed by visual inspection of host symptoms. In addition, the nearly complete genome of PLRV and the complete or partial genome sequence of multipartite virus segments have been identified. Besides the three major potato viruses that BLASTn analysis revealed were present in our samples, BLASTx analysis revealed some reads are derived from other potato viruses, such as potato virus V (PVV), Andean potato latent virus (APLV), and tomato chlorosis virus (ToCV), which are not frequently reported in potato field screenings in Egypt. Other microbial agents, such as bacteria and fungi, were also identified in the examined sample sequences. Some mycovirus sequences derived from ourmia-like viruses and *Alternaria alternata* chrysovirus were also identified in sample S4, confirming the complexity of the potato microbiome under field conditions.

**Conclusion:**

NGS quickly and accurately identifies potato plant viruses under field conditions. Implementing this technology on a larger scale is recommended to explore potato fields and imported plants, where symptoms may be absent, unspecific, or only triggered under certain conditions.

**Supplementary Information:**

The online version contains supplementary material available at 10.1186/s43141-023-00561-2.

## Background

Potato is considered to be the third most important crop plant for human consumption, following rice and wheat [1]. Various pathogens, including viruses, drastically decrease potato yield and quality, threatening an important food resource. Over 50 viruses and one viroid have been reported to infect potatoes worldwide [2–4]. However, only a limited number of those viruses are known to cause major global losses. As potato is a vegetatively propagated crop, it is prone to accumulating viruses in tubers from one generation to the next. Successful crop protection necessitates the use of adequate detection and identification methods. Visual inspection is usually unreliable because the symptoms may be mild or could be masked by other factors [5]. As a result, more accurate viral detection and identification approaches are required.

Worldwide, PVY is the most economically important virus, followed by PLRV. Tuber yield losses are high by either viral infections and can reach up to 80% when combined with other viruses [3]. The symptoms are exacerbated due to the synergistic effects of mixed potato virus infections. Aside from reducing yield, numerous viruses can cause revenue losses by affecting potato quality, especially by causing surface and internal tuber necrosis. For instance, some PVY strains can induce necrotic symptoms and are associated with the induction of “potato tuber ringspot disease.” Similarly, PLRV can occasionally cause “net necrosis” of the tuber vascular system [6].

Research on potato viruses has gained great attention in the USA and many European countries due to their impact on producing these economically important crop plants [7]. On the other hand, in Africa, there are only a few published studies about viruses that infect potatoes [3]. Moreover, the identification of potato viruses in most of these studies is routinely carried out using antibody affinity assays such as enzyme-linked immunosorbent assay (ELISA) and molecular techniques like reverse transcription polymerase chain reaction (RT-PCR), which are more frequently applied for large-scale testing. Recent techniques rely on the simultaneous detection of multiple viruses (multiplexed methods). Furthermore, in recent years, the next-generation sequencing (NGS) technology for virus detection has been the focus of many studies [8]. NGS has been used to identify viruses infecting many crops including garlic (*Allium sativum*), pepper (*Capsicum annuum*), grapevine (*Vitis vinifera*), potato (*Solanum tuberosum* L.), and tomato (*Solanum lycopersicum*) [9–13]. NGS has improved abilities to detect and characterize known and novel viruses as it is a hypothesis-free technology and has allowed exploring host-virus interaction [11, 14]. The few prior attempts to identify potato viruses using second- and third-generation sequencing approaches demonstrated that these techniques could be applied accurately and practically, particularly in the assembly of whole viral genomes and the differentiation of virus genotypes. For instance, a recent study revealed the possible differentiation of five PVY genotypes using nanopore sequencing. Moreover, the full genome of some potato viruses, such as potato virus X (PVX), potato virus S (PVS), and PLRV, was successfully assembled [15].

The current study was conducted on three field-collected potato leaf samples from three different potato fields in the same governorate to set the foundation for larger-scale detection of potato virome in Egyptian agricultural fields. Although NGS technology is widely utilized in Egypt in the fields of medicine [16, 17], archaeology [18], and zoology [19], it has never been applied to study plant viruses. To the best of our knowledge, this study is the first application of NGS to explore plant viruses in any Egyptian field.

## Methods

### Samples collection

Twenty Glatika cultivar (60–70 days old) of *Solanum tuberosum* (potato) plant leaf samples were collected from 10 potato farms in El Beheira Governorate, Egypt. A specialist from the relevant agricultural research center authorized the collection of the potato leaf samples. The samples were collected in the winter season of 2020 and transferred to the laboratory in RNAlater (AM7021, Ambion, Life Technologies) to preserve RNA integrity. One gram of fresh plant leaves (wiped with tissue to remove dust) was submerged in 5–10 mL of RNALater and stored at 4 °C. Virus-like symptoms were visually observed in all of the collected samples. In general, the symptoms included the following: severe mosaic, vein clearing, yellowing, chlorotic spots and rugosity, stunting and upward curling of leaves, yellow flecks on foliage, and calico symptoms. Three asymptomatic samples (control) were consequently collected for additional testing by RT-PCR.

### RNA-Seq library preparation and sequencing

The total RNA was prepared from the preserved plant leaf materials stored in RNAlater using QIAzol Lysis Reagent (Qiagen), following the manufacturer’s instructions. The integrity of the RNA was verified using Agilent Technologies 2100 Bioanalyzer. Based on the RNA integrity, three samples (S3, S4, and S6) were selected for sequencing. For RNA-Seq libraries preparation, ribosomal RNA was removed using Ribo-Zero™ (Plant Leaf) (Illumina Inc., San Diego, CA, USA) and combined with the TruSeq Stranded Total RNA LT Sample Prep Kit plant (Illumina Inc., San Diego, CA, USA) to capture both coding and noncoding RNA species. Illumina NovaSeq 6000 sequencer platform was used to sequence the pooled, paired-end (read size: 100 bp) libraries. Library construction and sequencing were conducted at Macrogen, Inc. Seoul, South Korea, https://dna.macrogen.com/. The three RNA samples S3, S4, and S6 generated 60,640,920, 55,359,172, and 60,700,498 reads, respectively.

### Bioinformatics analysis of the obtained RNA-Seq reads

The quality of the obtained raw reads was checked before the analysis using FASTQC. De novo transcriptome assembly was performed using the Trinity software package: https://github.com/trinityrnaseq/trinityrnaseq. Briefly, paired-end FASTQ reads were submitted to the Trinity software using default parameters, and the output of this analysis resulted in assembled FASTA sequence files for each of the three RNA-Seq samples (S3, S4, and S6). Each FASTA file containing assembled contigs was used in downstream analysis to identify viral sequences.

### Identification of viral sequences using VirusDetect software

The three assembled contig files were analyzed using the VirusDetect software package available through http://bioinfo.bti.cornell.edu/tool/VirusDetect/. VirusDetect provides an automated pipeline for efficient virus detection [20]. The default parameters of VirusDetect were applied. Furthermore, group 248 U97 (plants) was the selected database for BLAST analysis. The *Solanum tuberosum* isolate E4-63 genome was uploaded and selected as the host genome for filtration of the host reads. Each virus identified by VirusDetect (BLASTn) was further checked using BLASTn tool available through the NCBI. Briefly, the contig sequence(s) that were mapped to the specific virus genome identified by the program were blasted manually against the nucleotide database (NCBI). BLASTx analysis available through VirusDetect was used to identify other possible viruses known to infect potatoes according to Kreuze et al. (2020) [3].

### RNA-Seq reads analysis using Taxonomer software

The Taxonomer software was used to analyze the reads of the three sequenced samples. Taxonomer is a *k-mer*-based interactive metagenomics sequence analysis tool available through the University of Utah’s Center for Genetic Discovery in Salt Lake City, USA. It classifies each read into one of eight categories: human, bacteria, viruses, phages, fungi, ambiguous, unknown sequences, and PhiX (used as a control for Illumina sequencing runs) [21]. The complete analysis mode was used to screen all reads in each dataset.

### Validation for three major potato viruses

RT-PCR-based screening was performed to check the presence/absence of three major potato viruses in the total RNA of the three samples used to prepare RNA-Seq libraries. In addition, 17 potato symptomatic leave samples collected simultaneously were also used for the PCR analysis. As a control, three asymptomatic potato samples were included in this test. The screening was performed for AMV, PLRV, and PVY. Briefly, following the manufacturer’s instructions, total RNA was extracted using the GeneJET Plant RNA Purification Mini Kit (ThermoFisher Scientific, USA), followed by RNA quality and quantity check using NanoDrop®ND-1000 spectrophotometer (ThermoFisher Scientific, USA). Thermo Scientific™ RevertAid™ First-Strand cDNA Kit (Thermo Scientific, USA) was used in accordance with the manufacturer’s instructions for cDNA synthesis. Only 2.5 µl of the produced cDNA and specific virus primers were added to the DreamTaq Green PCR Master Mix (2X) for PCR. RT-PCR was performed using the primers and programs listed in Supplementary Table 1. PVY samples were further differentiated into genotypes using multiplex reverse transcription polymerase chain reaction assay as described by [22]. Sanger sequencing was conducted on RT-PCR-positive samples to confirm the identity of the infecting virus.

### Phylogenetic analysis of coat and movement proteins of major identified viruses

The entire amino acid sequence of PLRV coat protein was deduced in samples S4 and S6 using the ExPASy protein translation program https://web.expasy.org/translate/. Furthermore, the entire amino acid sequence of AMV movement protein was inferred in all three samples. Therefore, these sequences were used to infer the phylogenetic relationships with other members of the respective viral family, which were determined by BLASTp (NCBI). The ClustalW program (default parameters) was used to generate multiple sequence alignments for the protein sequences. The maximum likelihood method with 1000 bootstraps was combined for tree construction available through the MEGA7 program.

## Results

### RT-PCR detection of major putative potato viruses

The presence/absence of three major potato viruses was detected using RT-PCR in the three RNA-sequenced samples (S3, S4, and S6) and 17 other symptomatic samples collected from the same fields simultaneously. Twenty samples (Table 1) were infected with AMV, 17 with PLRV, and 12 with PVY (Supplementary Fig. S1a, b, and c). AMV, PLRV, and PVY were detected in the three RNA-sequenced samples (S3, S4, and S6).

**Table 1:** RT-PCR detection results of three major potato viruses in the collected potato leaf samples collected from Kom Hamada-Al-Beheira, Egypt

**Code**	**Sample no**	**Location**	**PVY**	**PLRV**	**AMV**
S1	1	Land 1-Kom Hamada-Al-Beheira	+	+	+
S2	2	Land 1-Kom Hamada-Al-Beheira	+	+	+
S3	3	Land 1-Kom Hamada-Al-Beheira	+	+	+
S4	4	Land 1-Kom Hamada-Al-Beheira	+	+	+
S5	5	Land 2-Kom Hamada-Al-Beheira	+	+	+
S6	6	Land 2-Kom Hamada-Al-Beheira	+	+	+
S7	7	Land 2-Kom Hamada-Al-Beheira	+	+	+
S8	8	Land 3-Kom Hamada-Al-Beheira	+	+	+
S9	9	Land 4-Kom Hamada-Al-Beheira	+	+	+
S10	10	Land 4-Kom Hamada-Al-Beheira	-	+	+
S11	11	Land 5-Kom Hamada-Al-Beheira	-	+	+
S12	12	Land 5-Kom Hamada-Al-Beheira	-	+	+
S13	13	Land 6-Kom Hamada-Al-Beheira	-	+	+
S14	14	Land 6-Kom Hamada-Al-Beheira	-	+	+
S15	15	Land 7-Kom Hamada-Al-Beheira	-	+	+
S16	16	Land 7-Kom Hamada-Al-Beheira	-	+	+
S17	17	Land 8-Kom Hamada-Al-Beheira	+	-	+
S18	18	Land 9-Kom Hamada-Al-Beheira	+	-	+
S19	19	Land 9-Kom Hamada-Al-Beheira	-	-	+
S20	20	Land 10-Kom Hamada-Al-Beheira	+	+	+
			12	17	20

**Fig. 1:**
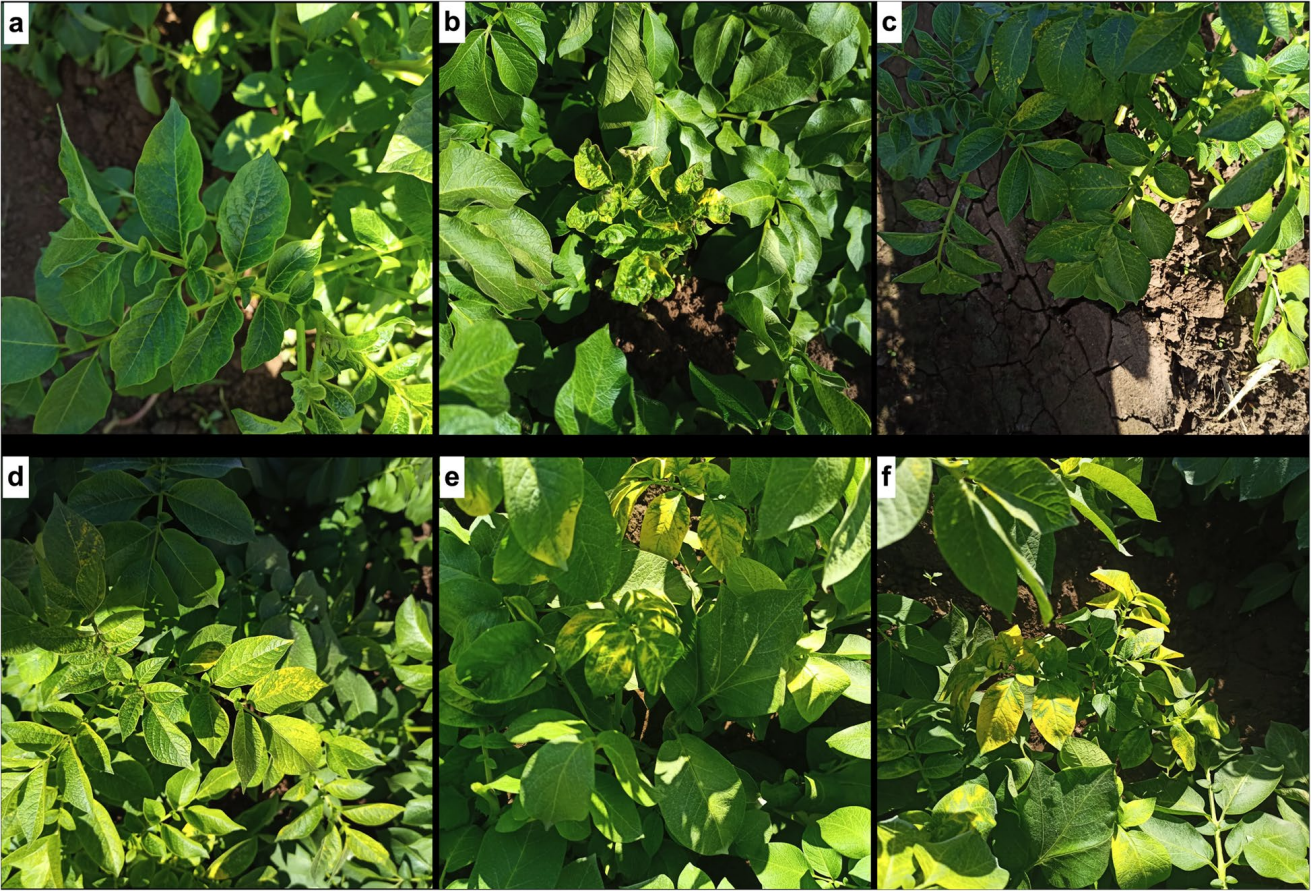
Potato leaf samples exhibiting virus-related symptoms such as light to dark green mosaic patterns (**a**), rugosity and curled leaf (**b**), chlorotic spots and vein clearing (**c**), yellowing (**d**), and calico symptoms characteristic of AMV (**e** & **f**)

For the symptomatic samples, virus-like symptoms were visually observed. However, there were variations in the appearance and strength of the symptoms. Symptoms on infected potato leaves included severe mosaic, vein clearing, yellowing, chlorotic spots, and rugosity symptoms, characteristic of PVY^N^ strains (Fig. 1a–c). Moreover, we noticed stunting and upward curling of leaves characteristic of PLRV, yellow flecks on foliage, and calico symptoms characteristic of AMV (Fig. 1). Overall, PVY and AMV symptoms were more dominant than PLRV symptoms. Examination of three asymptomatic potato leaves (control) revealed that despite the absence of obvious symptoms, analysis of the RNA from these plants using RT-PCR demonstrated the presence of PLRV (Supplementary Fig. S2).

### Potato viruses’ identification by RNA-Seq

Following de novo assembly of the RNA-Seq reads using Trinity, VirusDetect-based BLASTn analysis revealed the presence of viruses from four genera: *Alfamovirus*, *Polerovirus*, *Potyvirus*, and *Solendovirus* in examined samples. The analyses of the assembled trinity contig sequences derived from S3, S4, and S6 samples after filtration of host reads against the VirusDetect plant viruses database matched sequences of 21, 26, and 30 different plant viral sequences as listed by different accession numbers in the database. The accession numbers, the coverage, identity percentage, and number of assembled contigs that shared similarity with these viruses are listed in Supplementary Tables 2, 3, and 4. Based on VirusDetect BLASTn analysis, AMV was the most prevalent in the three tested samples. Similarly, tobacco vein-clearing virus (TVCV), not previously known as a potato crop virus, was identified in all samples. The PLRV, PVY, and red clover bacilliform virus (RCBV) were identified only in two of the three samples. Specifically, the former two were detected in S4 and S6, while RCBV was detected in S3 and S6 samples.

The identity percentage of the contig sequences identified in our samples with TVCV (NCBI accession number AF190123) ranged from 82.87 to 86.87%. The RCBV is a new member of the genus *Badnavirus* [23, 24]. It was isolated from *Trifolium pratense* plants, where it dominates in the phloem tissue inducing plant dwarfing and mosaic symptoms [23]. This virus was not reported from potato plants before. The identity percentage of the contig sequences from our analysis with RCBV (NCBI accession number JX069965) ranged from 82.52 to 84.12%. Interestingly, BLASTn search of AF190123 and JX069965 complete genome sequences against the NCBI database demonstrates that these viral genomes share similarities with TVCV, RCBV, *Solanum tuberosum* sequences, and other dicot plants. It has previously been reported that viral genomes integrate into plant chromosomes, which assists in genome evolution [25]. Therefore, whether TVCV and RCBV represent an actual active viral infection in our potato samples or reflect the presence of these viral integrated sequences in the host plant needs further investigation.

In addition to the viruses mentioned above, BLASTx analysis identified some reads that are derived from other potato viruses, namely potato virus V (PVV) (Potyviridae), Andean potato latent virus (APLV) (Tymoviridae), tomato chlorosis virus (ToCV) (Closteroviridae), tomato leaf curl Ghana virus (ToLCV) (Geminiviridae), cucumber mosaic virus (CMV) (Bromoviridae), and cauliflower mosaic virus (CaMV) (Caulimoviridae). The coverage, identity percentage, and number of contigs for these viruses are listed in Supplementary Table 5. Future field screenings are needed to verify the presence and abundance of these potato viruses, as none of them was routinely screened for in previous knowledge-based field examinations conducted in the Egyptian fields.

### Complete and partial genome sequences of potato viruses

**Fig. 2:**
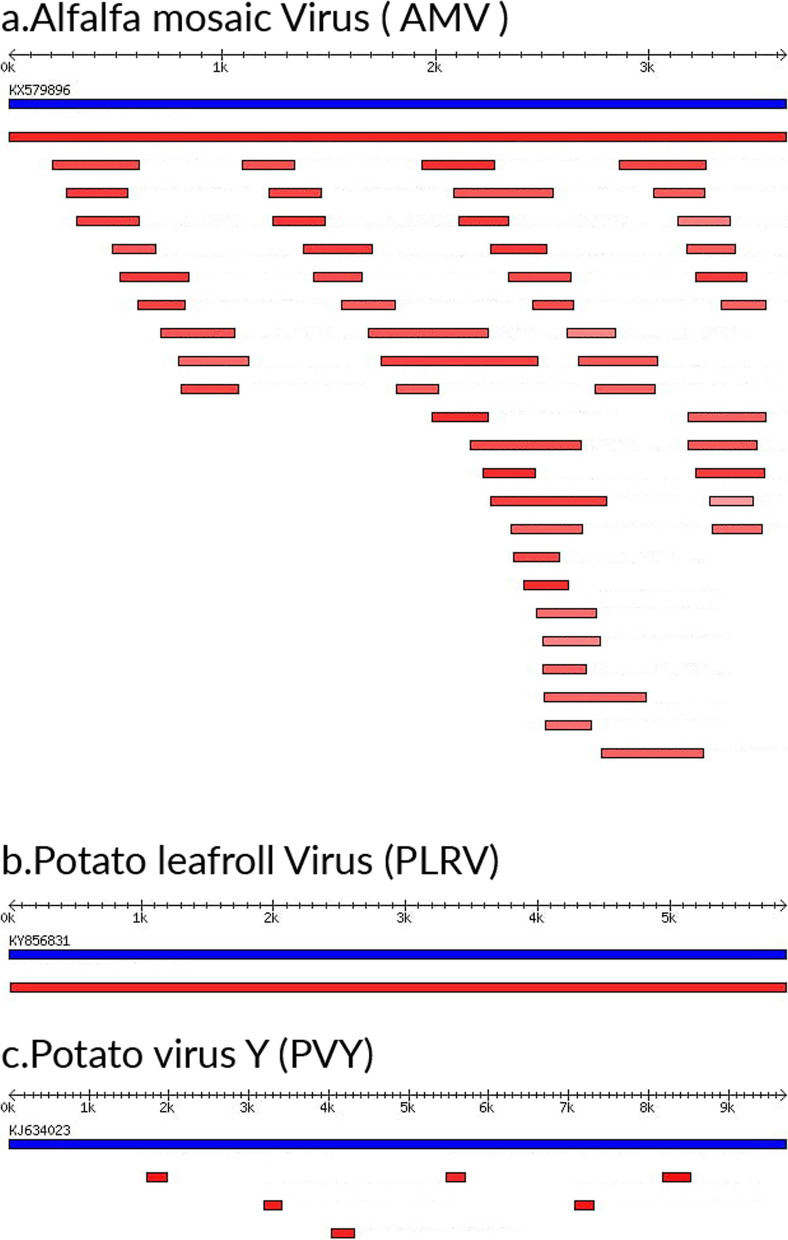
Major potato plant viruses identified by VirusDetect program BLASTn tool. **a–c** Schematic representation of the assembled contigs from a sample that can be aligned to a reference potato viral genome. The reference viral full or partial genome sequence NCBI accession number to which significant similarity is detected (in blue) and the contigs assembled from the sample (in red). The sample’s contigs color varies from light to dark red color based on identity percentage, where the red color intensity is directly proportional to identity percentage

The Trinity-based de novo assembly of the RNA-Seq reads generated complete or nearly complete genomes of PLRV. In sample S6, a single contig of 5881 bp in size was obtained (Fig. 2). Alignment of this contig sequence against VirusDetect plant viral genomes database revealed that it had 97.19% nucleotide identity to that of PLRV (Argentina: Tupungato, Mendoza) genomic RNA (NCBI accession number KY856831). A similar PLRV variant was detected in sample S4 (96.41% identity to the Argentinean strain) along with three other variants that shared similarity with three other PLRV strains in the database (NCBI accession numbers: KX712226, MT537598, D13953) (Supplementary Tables 2, 3, and 4). Moreover, complete and partial genome sequences of some genomic segments in multipartite viruses such as AMV were detected in the three samples. For instance, AMV genomic segments number one, two, and three encode the replicase (P1 and P2) and movement and CP proteins, respectively (Fig. 2).

### Mycoviruses identified sequences

**Fig. 3:**
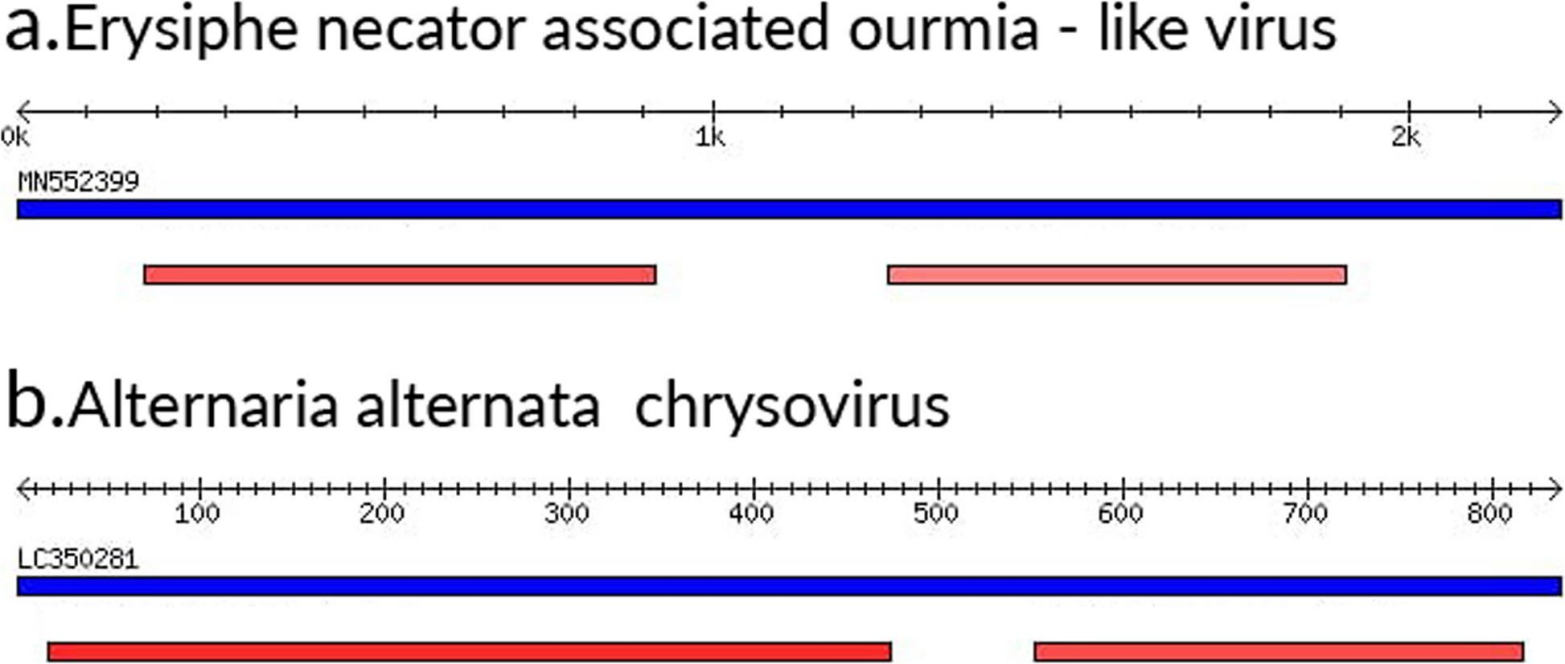
Examples of mycoviruses identified by VirusDetect program BLASTn tool in potato sample S4. **a**–**b** Schematic representation of two mycoviruses identified in sample S4. The assembled contigs from sample S4 that can be aligned to a reference mycoviral genome. The reference mycoviral genome sequence NCBI accession number to which significant similarity is detected (in blue) and the contigs assembled from sample S4 (in red). The sample’s contigs color varies from light to dark red color based on identity percentage, where the red color intensity is directly proportional to identity percentage

In addition to the viruses mentioned above, sample S4 was unique in that it contained some mycovirus sequence reads. For example, using the BLASTn tool of VirusDetect, the following mycoviruses were identified: *Plasmopara viticola* lesion-associated ourmia-like virus (Botourmiaviridae), *Bremia lactucae*-associated ourmia-like virus (Botourmiaviridae), soybean leaf-associated *Ourmiavirus* (Botourmiaviridae), *Erysiphe necator*-associated viruses (Botourmiaviridae), and *Alternaria alternata* chrysovirus (Chrysoviridae) (Fig. 3; Supplementary Table 3). Overall, many of these mycoviruses are taxonomically related and may represent quasi-species.

### Identification of viruses and other microbes using the Taxonomer program

To determine the general pattern of the three potato plant samples S3, S4, and S6 regarding viruses and other microbes, we applied the “Taxonomer” program. The full analysis mode of the three potato samples’ raw paired-end FASTQ files revealed that the dominant potato viruses identified by VirusDetect in our samples (i.e., AMV, PLRV, and PVY) were further confirmed by Taxonomer; however, unlike what VirusDetect reported, sample S3 was found to be infected by PLRV and PVY but in very low abundance.

In addition to detecting viral sequences, Taxonomer identified other important microbial agents in our sample sequences, such as bacteria and fungi, which coexist with viral infections. For example, the top three bacterial species identified in sample S3 were *Leptolyngbya* sp., *Paenibacillus* sp., and *Thiomicrospira thermophila*. The top three bacterial species detected in sample S4 were *Leptolyngbya* sp., *Thiomicrospira thermophila*, and *Nitrosospira* sp., while the top three bacterial species detected in sample S6 were *Leptolyngbya* sp., *Chroococcidiopsis* genus, and *Paenibacillus* sp. The *Leptolyngbya* sp., a genus of cyanobacteria, is therefore regarded as the most prevalent in all samples. Furthermore, the fungal infection percentage in our samples was minimal (ranging from 0.03 to 0.22%). However, no specific fungal pathogen had a high relative abundance in any sample. This result reflects the complex pathogen-pathogen and host–pathogen interactions occurring in nature. Overall, more PCR validation and quantitation methods are needed to confirm the presence and amount of these microbes and their possible association with control strategies.

### Phylogenetic analysis of viral coat and movement proteins

**Fig. 4:**
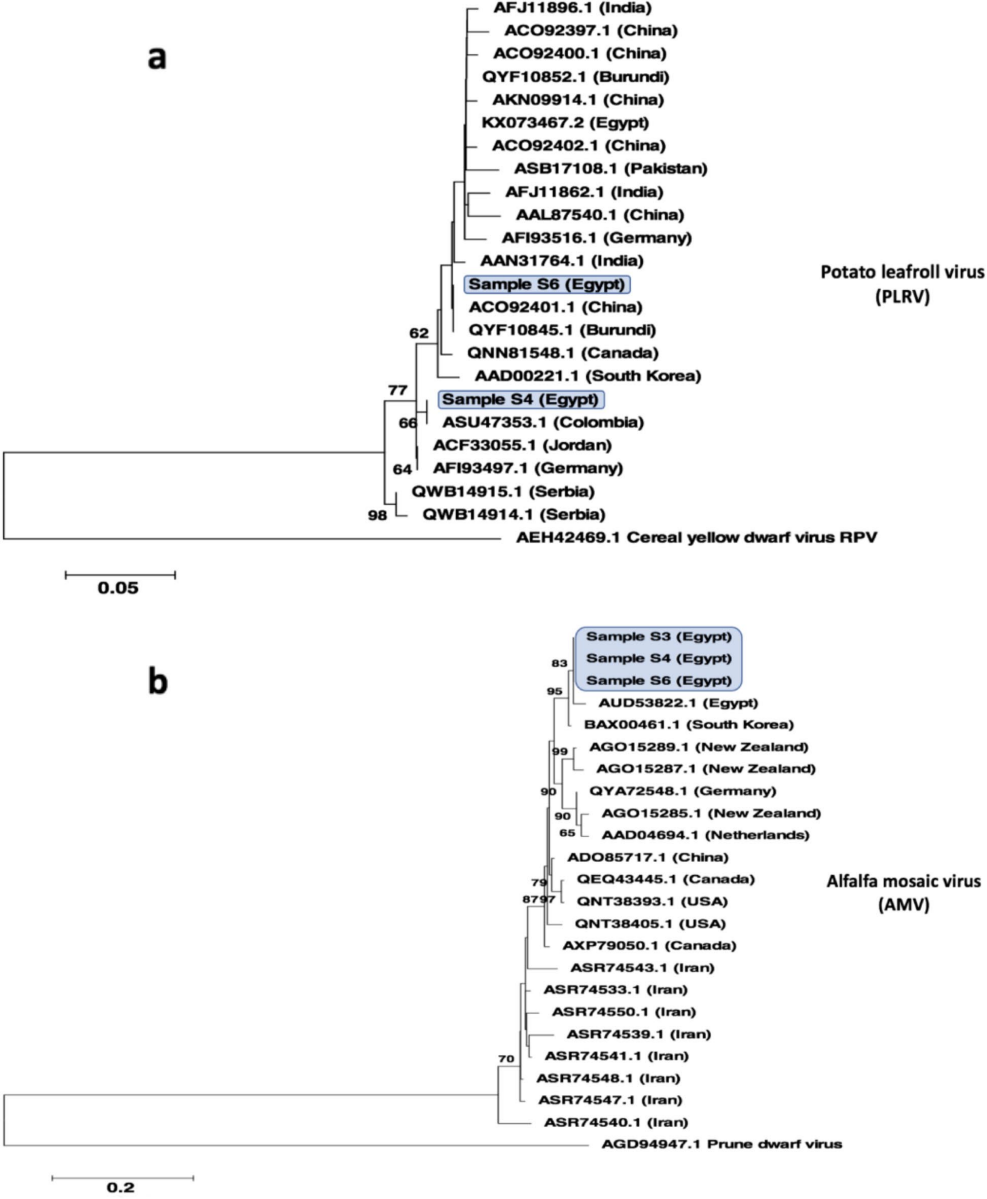
Study of the evolutionary relationships between **a** potato leafroll virus (PLRV) and **b** alfalfa mosaic virus (AMV) variants identified using the coat and movement protein sequences, respectively. The phylogenetic trees were constructed based on the amino acid sequence of the listed viral structural genes and by comparison to closely related homologs. The trees were built using the maximum likelihood method combined with bootstrapping (1000 replicates). The analyses were conducted using MEGA7. Bootstrap values for nodes greater than 50% are shown in the tree

Phylogenetic analysis was conducted using the coat protein’s amino acid sequences of two PLRV variants identified in samples S4 and S6. The phylogenetic analysis based on the maximum likelihood method with bootstrap replicate value 1000 revealed that the PLRV variant identified in sample S6 was quite different from sample S4 and was grouped in a monophyletic clade with ACO92401.1 and QYF10845.1 PLRV variants from China and Burundi, respectively (Fig. 4a). Furthermore, the tree demonstrates that a previous PLRV variant isolated in 2017 from Egypt (NCBI accession number KX073467.2) was quite different from the current PLRV identified in this study.

The phylogenetic analysis of the movement protein amino acid sequence of three AMV variants based on the maximum likelihood method with bootstrap replicate value 1000 revealed the close phylogenetic relationship between the three AMV variants, at least based on this protein analysis. The three variants were grouped in a single monophyletic clade (Fig. 4b). A previously isolated AMV variant in 2017 from Egypt (NCBI accession number AUD53822.1) was highly related to the currently identified variants.

## Discussion

Because of the regular use of PCR, RT-PCR, and antisera for plant virus detection, many of these viruses are either underestimated or undetected. On the other hand, the application of metagenomics and metatranscriptomics in virome studies of diverse environmental samples proves that the field of virology has only uncovered less than 1% of the total viral diversity. The rate of virus discovery using new omics technology is way higher than all other approaches combined [26, 27]. The virome for many important crops was discovered recently using these new technologies. Identifying plant virome is essential to understand virus biology, diversity, and disease etiology [28]. Therefore, in the current study, RNA-Seq technology was applied on three field-collected potato host’s leaf samples to provide a foundation for future bigger-scale detection of potato virome in Egyptian agricultural fields.

Sequence analysis identified some of the most reported potato viruses in Egypt and other African countries. RNA-Seq data revealed that the three sequenced potato leaf samples (S3, S4, and S6) were infected by several viruses. Some important potato plant viruses were detected, namely, AMV, PLRV, and PVY. Previous ELISA and RT-PCR-based studies have also reported the presence of AMV, PLRV, and PVY in Egyptian potato fields [29–31] and other African countries. For instance, surveys in Kenya [32], Tanzania [33], South-West Uganda, and Sudan [34] identified multiple potato virus infections. PLRV and potato virus A (PVA) were prevalent in Kenya, with 71% and 75% detection rates in screened potato samples, respectively. PLRV and potato virus S (PVS) were the most common viruses in Tanzania, with 39% and 55% detection rates, respectively [3]. In 2014, screening for potato viruses in South-West Uganda revealed that potato virus X (PVX) and PLRV were the most prevalent, followed by PVY and potato virus M (PVM). In Sudan, however, AMV and beet curly top virus (BCTV) were the most prevalent viruses. Intriguingly, the African studies revealed that mixed infections were common, with only 2.4% of tubers being virus-free [3]. Internationally, field surveys in Colombia revealed the incidence of PVY as 13.3 to 80% [35]. In Peru, PVY was shown to be present in ~ 43% of tested samples [36]. According to a study conducted in Bangladesh’s key potato-growing regions, the prevalence of PLRV, PVX, PVY, PVS, potato virus H (PVH), potato aucuba mosaic virus (PAMV), and PVM ranged from 2 to 8.33% [37]. PVY, PLRV, and PVM were the most common viruses in India, accounting for 44.26%, 42.62%, and 27.86%, respectively [38]. Recent surveys in Brazil have found PVX levels as high as 10% and PVS levels as 20% in imported potato seeds [39].

In addition, the analysis revealed the presence of other plant viruses that are not normally known to infect potatoes according to Kreuze et al. (2020) [3]. Specifically, TVCV and RCBV were identified. TVCV and RCBV are both pararetroviruses, known as endogenous viruses, and become integrated into their host genome. When exposed to factors such as abiotic stress, some of these viruses give rise to infectious episomal forms of the virus [24]. Such behavior prevents the detection of these viruses using traditional techniques such as EM. Therefore, NGS-based techniques could help in detecting such rare viral sequences.

Other potato viruses detected in this study, for example, PVV, APLV, ToCV, TolCV, CMV, and CaMV, on the other hand, are not frequently reported in potato field screenings in Egypt based on traditional techniques. Only one study reported the presence of CMV-infected potato in Egypt Assiut governorate [40]. ToCV has been linked to high incidence levels in tomato crops, but not in potato crops [41]. Planting alternate hosts in the same field or surrounding fields, such as potato with tomato, allows the virus to be passed from tomato to potato plant, which is why those viruses might also be present in the three examined field samples.

Moreover, the simultaneous presence of multi-viral infections was noticed in the tested samples (Table 1). Multiple infections result in various virus–virus interactions, which can be classified as synergism, neutralism, or antagonism. Studying virus–virus interactions in plants could be key to understanding viral pathogenesis and evolution. Further studies must be performed to study the interactions that occur between those viruses in potato plants. In this regard, despite the absence of visible symptoms, analysis of the RNA using RT-PCR revealed that PLRV was present in all three of the asymptomatic (control) potato plants (Supplementary Fig. S2). This finding consequently supports the possibility that the PLRV infection is new or of very low load that it has not yet manifested any apparent signs. The combined infection, on the other hand, might have caused a synergistic interaction that accelerated the emergence of PLRV-specific symptoms, including stunting and upward curling (Fig. 1). With a few exceptions of antagonism, the combined infection is expected to have a synergistic effect on at least one of the coexisting viruses [42]. It was observed that the mixed infection was frequently to blame for the sharp increase in disease symptoms and outbreaks [42–44]. Moreover, the different viral variants may induce different symptoms. In this regard, the phylogenetic analysis revealed that the PLRV variant identified in S4 and S6 samples was quite different from the previously detected variant in 2017 from Egypt (NCBI accession number KX073467.2) (Fig. 4a).

Interestingly, some mycoviruses-related reads were identified in sample S4 (Fig. 3). According to Taxonomer analysis, the other samples S3 and S6 may harbor some fungi, however, by a less percentage. Specifically, samples S3, S4, and S6 had 0.06%, 0.22%, and 0.03% fungal load, respectively. We can hypothesize that the presence of fungi justifies the detection of mycovirus. Mycoviruses are viruses that infect fungi and are ubiquitous in nature. What role mycoviruses play in these multitrophic associations where plants, fungi, and mycoviruses coexist is still to be determined. One of the defined roles of these triple associations is to enhance the thermal tolerance of the system as detected in geothermal soils in US Yellowstone National Park, where the extreme environmental conditions dominate [27]. Understanding the role of mycoviruses in plant protection against pathogenic fungi could lead to new applications in plant protection strategies in the future.

In addition to mycoviruses, the dominance of cyanobacteria-associated reads from genus *Leptolyngbya* sp. in the three samples could explain the limited fungal load range (0.03 to 0.22%) detected in them. Recently, several studies have confirmed cyanobacteria’s role in controlling many fungal plant pathogens. Cyanobacteria, for example, are effective in combating potato brown rot disease caused by *Ralstonia solanacearum* [45], tomato gray mold disease caused by *Botrytis cinerea*, and *Rhizoctonia solani* diseases [46, 47]. As a result, because this study was conducted under the field conditions, where we observed an inverse correlation between *Leptolyngbya* sp. dominance, at least on the sequences level, and a sharp decrease in fungal load, further research into the antifungal properties of this cyanobacterial genus is recommended.

## Conclusions

The current study emphasizes the importance of adopting NGS on a broader scale for identifying potato viruses in various disciplines, notably for analyzing imported plants where symptoms may be absent, unspecific, or only triggered under specific conditions.

### Supplementary Information

**Additional file 1: Table 1.** RT-PCR programs used for the molecular detection of AMV, PLRVand PVY in collected potato samples. **Table 2.** Plant viruses identified by VirusDetect BLASTn alignment of the RNA-Sequencing reads derived from potato sample S3. **Table 3.** Plant viruses identified by VirusDetect BLASTn alignment of the RNA-Sequencing reads derived from potato sample S4. **Table 4.** Plant viruses identified by VirusDetect BLASTn alignment of the RNA-Sequencing reads derived from potato sample S6. **Table 5.** Other possible potato plant viruses detected by VirusDetect BLASTx alignment of the RNA-Sequencing reads derived from potato samples S3, S4 and S6.

**Additional file 2: Fig. S1.** Agarose gel electrophoresis of Reverse Transcription-Polymerase Chain Reaction (RT-PCR) amplicon products generated by (a) AMV, (b) PLRV and (c) PVY. Lane M, represents DNA molecular weight marker (100-1000 bp) (Thermo scientific). Lane 1-20, represents ~ 8 µl aliquots of amplified sample, primer pairs described in supplementary Table 1A. Specifically, (A) shows a 651 bp amplicon of the AMV coat protein (CP) gene, indicating that all tested samples were positive for AMV. (B) Seventeen samples were positive for PLRV and showed the 381 bp PLRV CP band. (C) Using the primer pairs for the identification of PVY infection (Lorenzen et al., 2006) only 12 samples showed positive identification of PVYNTN or PVYN:O (181 bp band), the presence of the 452 bp band, appears in only some but not all samples. **Fig. S2.** On top, images of collected asymptomatic potato plants (C1-C3) followed by electrophoretic mobility of DNA amplicons obtained by RT-PCR from total RNA of asymptomatic potato leave samples using AMV, PVY and PLRV primer pairs, respectively. Lane M: Molecular weight marker 100-1000 bp (Thermo Scientific). Despite the absence of obvious symptoms, analysis of the RNA from asymptomatic potato leaf samples showed the presence of the 381 bp coat protein (CP) band that is characteristic of PLRV.

## Data Availability

The datasets generated for this study can be found in the GEO database [https://www.ncbi.nlm.nih.gov/geo/; BioProject ID PRJNA743866].

## Abbreviations

AMV: Alfalfa mosaic virus
NGS: Next-generation sequencing
PAMV: Potato aucuba mosaic virus
PLRV: Potato leafroll virus
PVH: Potato virus H
PVS: Potato virus S
PVX: Potato virus X
PVY: Potato virus Y
RCBV: Red clover bacilliform virus
RNA-Seq: RNA sequencing
TVCV: Tobacco vein clearing virus
