# Supplementary material for: Exploring virus presence in field-collected potato leaf samples using RNA sequencing
Source: J Genet Eng Biotechnol. 2023 Oct 20;21:106. doi: 10.1186/s43141-023-00561-2 (PMC10589165; doi:10.1186/s43141-023-00561-2)
Supplement: Supplementary file 2 — Additional file 2: Fig. S1. Agarose gel electrophoresis of Reverse Transcription-Polymerase Chain Reaction (RT-PCR) amplicon products generated by (a) AMV, (b) PLRV and (c) PVY. Lane M, represents DNA molecular weight marker (100-1000 bp) (Thermo scientific). Lane 1-20, represents ~ 8 µl aliquots of amplified sample, primer pairs described in supplementary Table 1A. Specifically, (A) shows a 651 bp amplicon of the AMV coat protein (CP) gene, indicating that all tested samples were positive for AMV. (B) Seventeen samples were positive for PLRV and showed the 381 bp PLRV CP band. (C) Using the primer pairs for the identification of PVY infection (Lorenzen et al., 2006) only 12 samples showed positive identification of PVYNTN or PVYN:O (181 bp band), the presence of the 452 bp band, appears in only some but not all samples. Fig. S2. On top, images of collected asymptomatic potato plants (C1-C3) followed by electrophoretic mobility of DNA amplicons obtained by RT-PCR from total RNA of asymptomatic potato leave samples using AMV, PVY and PLRV primer pairs, respectively. Lane M: Molecular weight marker 100-1000 bp (Thermo Scientific). Despite the absence of obvious symptoms, analysis of the RNA from asymptomatic potato leaf samples showed the presence of the 381 bp coat protein (CP) band that is characteristic of PLRV. [file 43141_2023_561_MOESM2_ESM.pdf]

Fig. S1. Agarose gel electrophoresis of Reverse Transcription-Polymerase Chain Reaction (RT-PCR) amplicon products generated by (a) AMV, (b) PLRV and (c) PVY. Lane M, represents DNA molecular weight marker (100-1000 bp) (Thermo scientific). Lane 1-20, represents ~ 8 µl aliquots of amplified sample, primer pairs described in supplementary **Table 1A**. Specifically, (A) shows a 651 bp amplicon of the AMV coat protein (CP) gene, indicating that all tested samples were positive for AMV. (B) Seventeen samples were positive for PLRV and showed the 381 bp PLRV CP band. (C) Using the primer pairs for the identification of PVY infection (Lorenzen et al., 2006) only 12 samples showed positive identification of PVY^{NTN} or $PVY^{N:O}$ (181 bp band), the presence of the 452 bp band, appears in only some but not all samples.

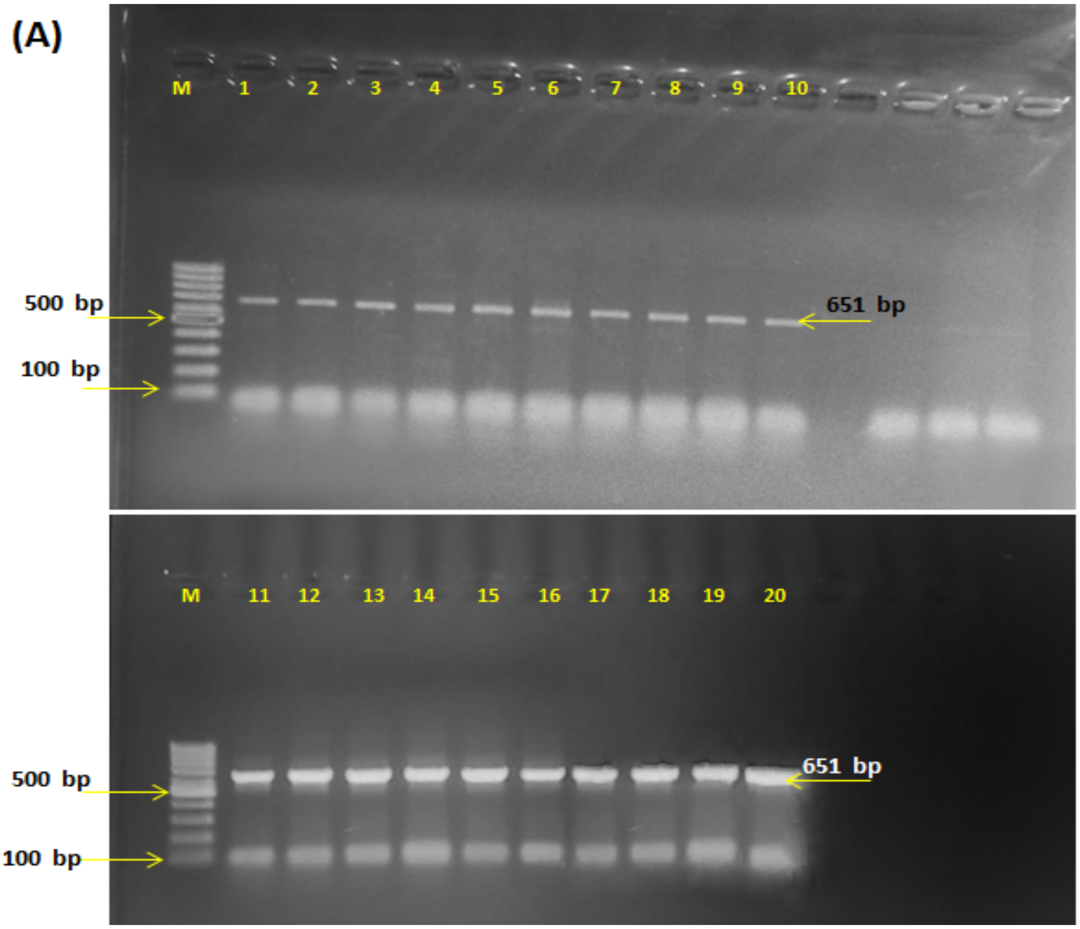

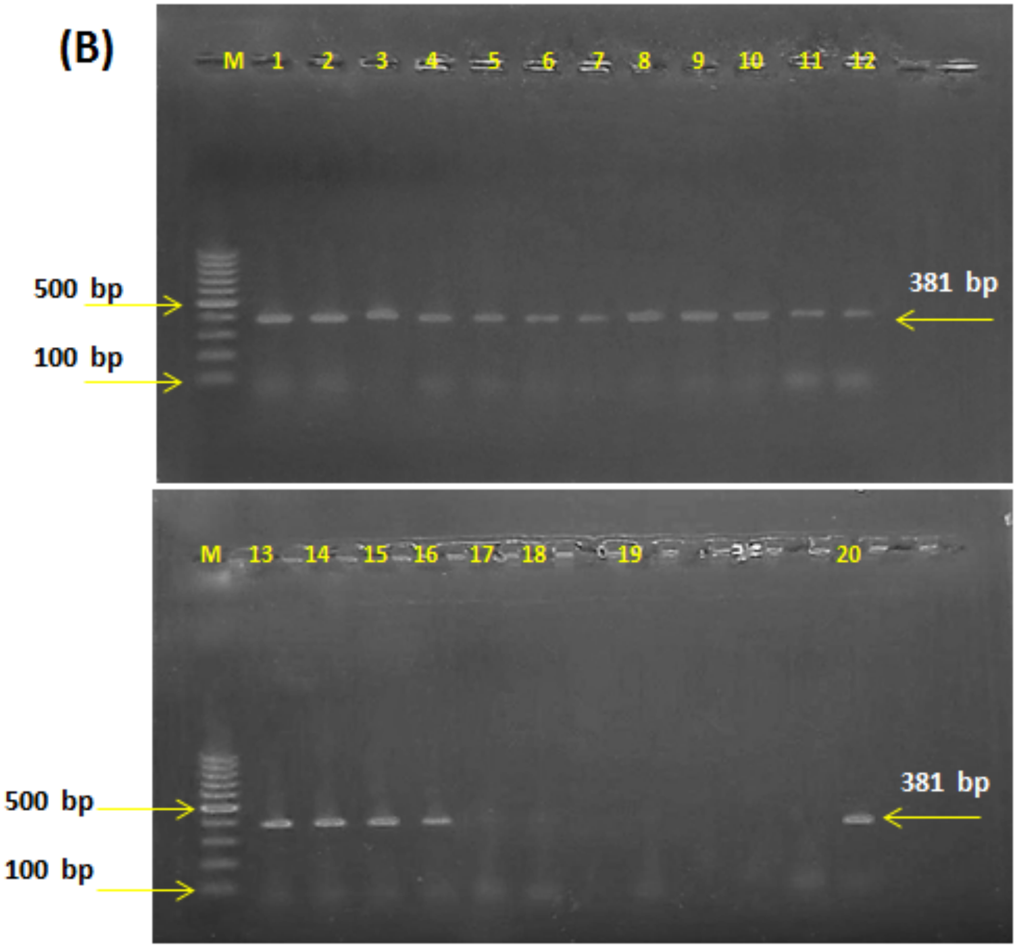
(B)
M 1 2 3 4 5 6 7 8 9 10 11 12
500 bp
100 bp
381 bp
M 13 14 15 16 17 18 19 20
500 bp
100 bp
381 bp

(C)

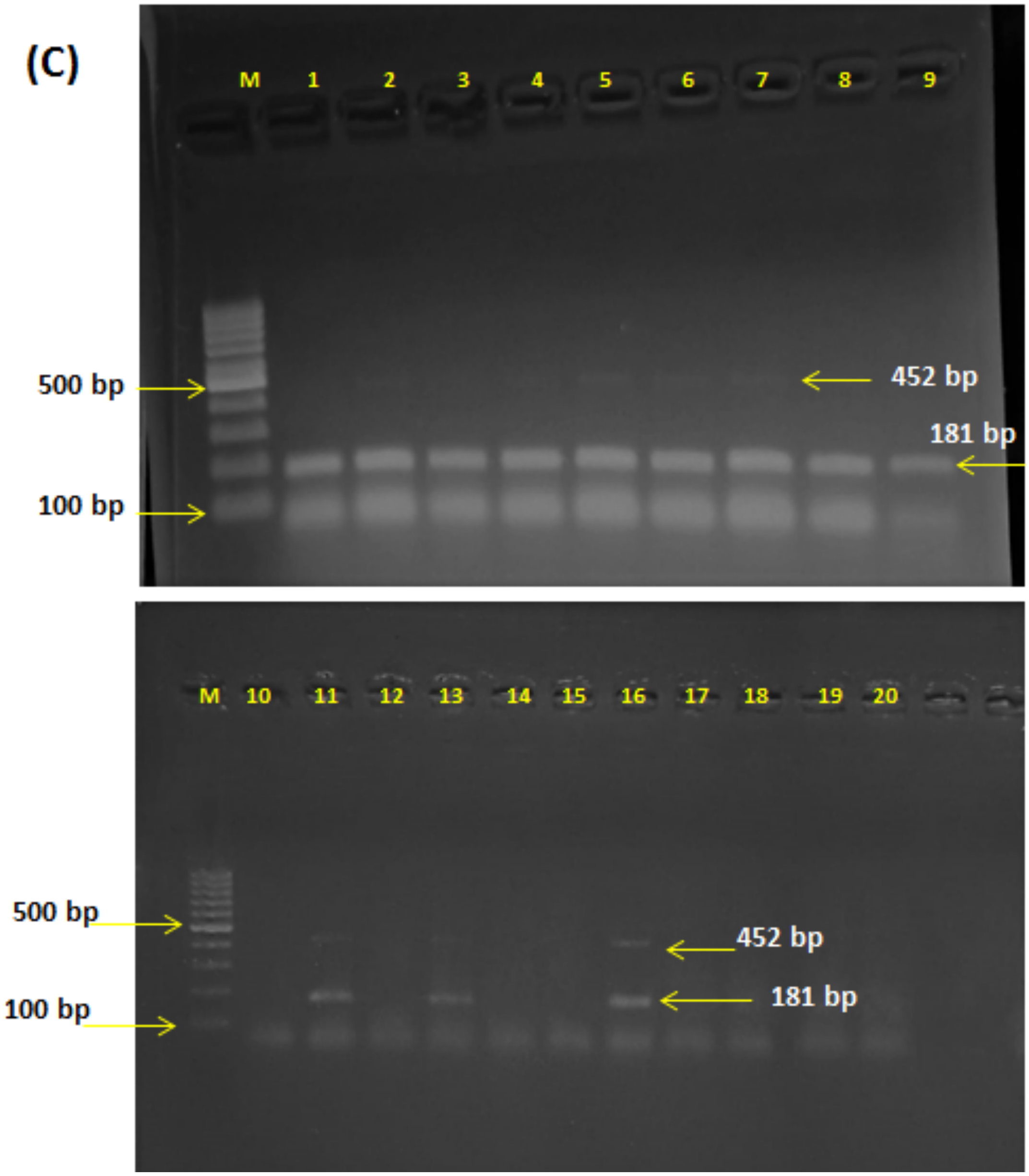
M 1 2 3 4 5 6 7 8 9
500 bp
100 bp
452 bp
181 bp
M 10 11 12 13 14 15 16 17 18 19 20
500 bp
100 bp
452 bp
181 bp

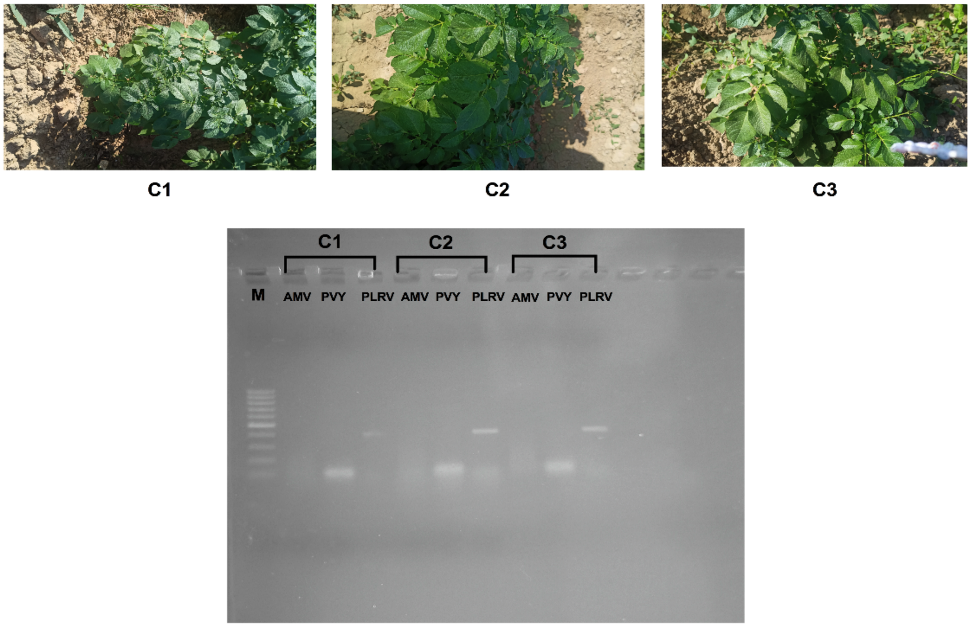

Fig. S2. On top, images of collected asymptomatic potato plants (C1-C3) followed by electrophoretic mobility of DNA amplicons obtained by RT-PCR from total RNA of asymptomatic potato leave samples using AMV, PVY and PLRV primer pairs, respectively. Lane M: Molecular weight marker 100-1000 bp (Thermo Scientific). Despite the absence of obvious symptoms, analysis of the RNA from asymptomatic potato leaf samples showed the presence of the 381 bp coat protein (CP) band that is characteristic of PLRV.
